# LigBuilder V3: A Multi-Target *de novo* Drug Design Approach

**DOI:** 10.3389/fchem.2020.00142

**Published:** 2020-02-28

**Authors:** Yaxia Yuan, Jianfeng Pei, Luhua Lai

**Affiliations:** ^1^Beijing National Laboratory for Molecular Sciences, State Key Laboratory for Structural Chemistry of Unstable and Stable Species, College of Chemistry and Molecular Engineering, Peking University, Beijing, China; ^2^Center for Quantitative Biology, Academy for Advanced Interdisciplinary Studies, Peking University, Beijing, China; ^3^Center for Life Sciences, Academy for Advanced Interdisciplinary Studies, Peking University, Beijing, China

**Keywords:** *De novo* design, Multi-target drug design (MTDD), multi-target drug optimization, Dual-functional inhibitors, LigBuilder

## Abstract

With the rapid development of systems-based pharmacology and poly-pharmacology, method development for rational design of multi-target drugs has becoming urgent. In this paper, we present the first *de novo* multi-target drug design program LigBuilder V3, which can be used to design ligands to target multiple receptors, multiple binding sites of one receptor, or various conformations of one receptor. LigBuilder V3 is generally applicable in *de novo* multi-target drug design and optimization, especially for the design of concise ligands for protein targets with large difference in binding sites. To demonstrate the utility of LigBuilder V3, we have used it to design dual-functional inhibitors targeting HIV protease and HIV reverse transcriptase with three different strategy, including multi-target *de novo* design, multi-target growing, and multi-target linking. The designed compounds were computational validated by MM/GBSA binding free energy estimation as highly potential multi-target inhibitors for both HIV protease and HIV reverse transcriptase. The LigBuilder V3 program can be downloaded at “http://www.pkumdl.cn/ligbuilder3/”.

## Introduction

For most of the twentieth century, drug discovery process was dominated by a reductionist “one disease, one target, one molecule” philosophy (Alcaro et al., 2019). Researchers and pharmaceutical industries around the world have been struggling to develop highly specific regulators against particular targets, which are generally expected to achieve higher potencies while reducing the risk of off-target related side effects (Eaton et al., 1995; Morphy and Rankovic, 2009; Hughes et al., 2011). Although successful drugs have been brought to market with this approach, new drug R&D aiming novel targets was noticeable slowdown and fewer drugs were approved over the last decades (Scannell et al., 2012; Ramsay et al., 2018), which implies the limitation and deficiency of previous single-target drug discovery strategy. Due to the complexity of biological network (Gerstein et al., 2012), disease usually involves multiple factors and biological pathways, so agents that directly interfere individual molecular targets often lack effectiveness at treating complex diseases (Brown and Superti-Furga, 2003; Kamb et al., 2007; Cavalli et al., 2008; He et al., 2016). Moreover, the upstream components of pathways have to be regulated if only one target is aimed at in a multiple pathology related disease, which is more likely to cause unexpected side effects. Consequently, researchers and pharmaceutical industries have been turning their attention to develop therapies that modulate multiple targets simultaneously (Reddy and Zhang, 2013; Zhang et al., 2017; Kumar and Sharma, 2018). Combination therapy and multi-target therapy were proposed to address this problem.

Combination drugs, which is defined as a concerted pharmacological intervention of multiple targets with several compounds, have been used increasingly to treat many types of diseases, such as viral and bacterial infection, cancer, hypertension, and atherosclerosis (Giles et al., 2014; Von Hoff et al., 2014; Blonde et al., 2015; Lu et al., 2018). Although the combination therapy is proposed to set up a new direction for drug discovery, it is not a new concept. In fact, using multi-component mixture extracted from natural products is a historical therapy in traditional medical treatments. Besides, the highly active antiretroviral therapy (HAART) (Lu et al., 2018), which is also known as the “AIDS cocktail,” has been the first-line anti-AIDS treatment since the end of last century (Bhatti et al., 2016). Many combination drugs have been launched to market and proved to be effective therapies for complex diseases, however, poor patient compliance has been raised especially in treatment of asymptomatic diseases such as hypertension (Eisen et al., 1990). An alternative way to simplify drug dosing is to mix multiple drug components into single co-formulated tablet, but different PK/PD property of each component may complicate the formulation and raise the risk of drug-drug interaction, and increase the risk and cost of such fix dose combinations strategy (Morphy and Rankovic, 2009).

Multi-target drug, which is defined as single compound that interacts with multiple targets simultaneously, has been paid much attention recently. Multi-target therapy is expected to be new and more effective medications for a variety of complex diseases even with relatively weak activity (Korcsmaros et al., 2007; Zimmermann et al., 2007). The uniform chemical component of multi-target drug will introduce lower risk of drug-drug interaction comparing with multi-components strategy. Moreover, although the discovery process of multi-target drug will be more complicated in the design and optimization stage due to the increased constraints from multiple targets, the risk and costs for the most expensive clinic trial stage are in principle similar with traditional single-target drug development. Consequently, many methods for multi-target ligand discovery were developed (Morphy et al., 2004; Zhan and Liu, 2009; Abdolmaleki et al., 2017; Zhang et al., 2017), such as multi-target QSAR (González-Díaz et al., 2006), fragment linker strategy (Morphy and Rankovic, 2006), framework combination (Morphy and Rankovic, 2006; Chen et al., 2011), and common pharmacophore based virtual screening and cross screening (Wei et al., 2008). Among them, framework combination and cross screening are both widely used approaches for discovering of multi-target lead (Morphy and Rankovic, 2005, 2009, 2010; Wu et al., 2012; Lepailleur et al., 2014; Bottegoni et al., 2016). Framework combination approach is based on the integration of multiple compounds via the fusion of common or similar sub-structure. Although the combined molecule from this approach is usually much smaller than directly linking two distinct structures with flexible chain, the median ligand efficiency is typically lower than general preclinical compounds which may lead to possible poor oral pharmacokinetics (Morphy and Rankovic, 2007). An alternative way is to screen multiple targets with the same compound library and select the consensus hints, namely, cross screening (Geppert et al., 2010). Although reported compounds derived by cross screening are better in ligand efficiency than that of framework combination approach, they are still statistically less efficient than general preclinical compounds. Considering the requirement of interacting with distinct binding sites, we are not surprising in the relative low ligand efficiency of multi-target compounds designed by the above methods (Morphy and Harris, 2012). Therefore, it is critical for multi-target compounds to be “highly integrated” that could make the most of each component group in multiple interactions. Moreover, the optimization of multi-target lead is far more complicated than that of single-target lead, because the “optimization landscape” of multi-target lead is no longer a simple stepwise “group-activity” profile in single-target lead optimization. The requirement of binding affinity balance for multiple binding will significantly reduce the available chemical space of the lead structure, as a result, stepwise optimization in multi-target optimization easily leads to “the blind alley,” namely, local minima. The increased dimensions in “optimization landscape” of multi-target lead optimization make the stepwise strategy less efficient, and implies that a more global and extensive structure sampling is necessary in optimization, which may be difficult to be achieved by manual work. It also suggests that a “one-step” design rather than routine “optimizing-bioassay” cycle is more suitable for multi-target drug discovery process. Therefore, the efficient discovery strategy of “highly integrated” ligand for unrelated targets remains challenging and a general strategy of multi-target rational drug design for dissimilar targets needs to be developed.

We developed an innovative multi-target design method, called LigBuilder V3, which enables the *de novo* design and molecular optimization algorithm to handle multiple targets. The chemical space exploration algorithm inherited from LigBuilder V2 (Yuan et al., 2011) has been upgraded to explore more sophisticated structure space of multi-target ligands. As we design the multi-target ligands from scratch with the consideration of multiple interactions of each component group, high ligand efficiency is expected to be achieved with this *de novo* design approach, which is very important for multi-target drugs. Multi-target lead optimization is also implemented in LigBuilder V3, which can help researchers to find possible multi-target optimization solutions. Furthermore, we apply an “ensemble linking” strategy to promote the efficiency of “fragment linking” algorithm and make it available in linking fragments for multi-target design, which is helpful in highly efficient recombination of known ligands and framework combination.

## Method and Algorithm

### Data Structure and Definition

**Figure 1:**
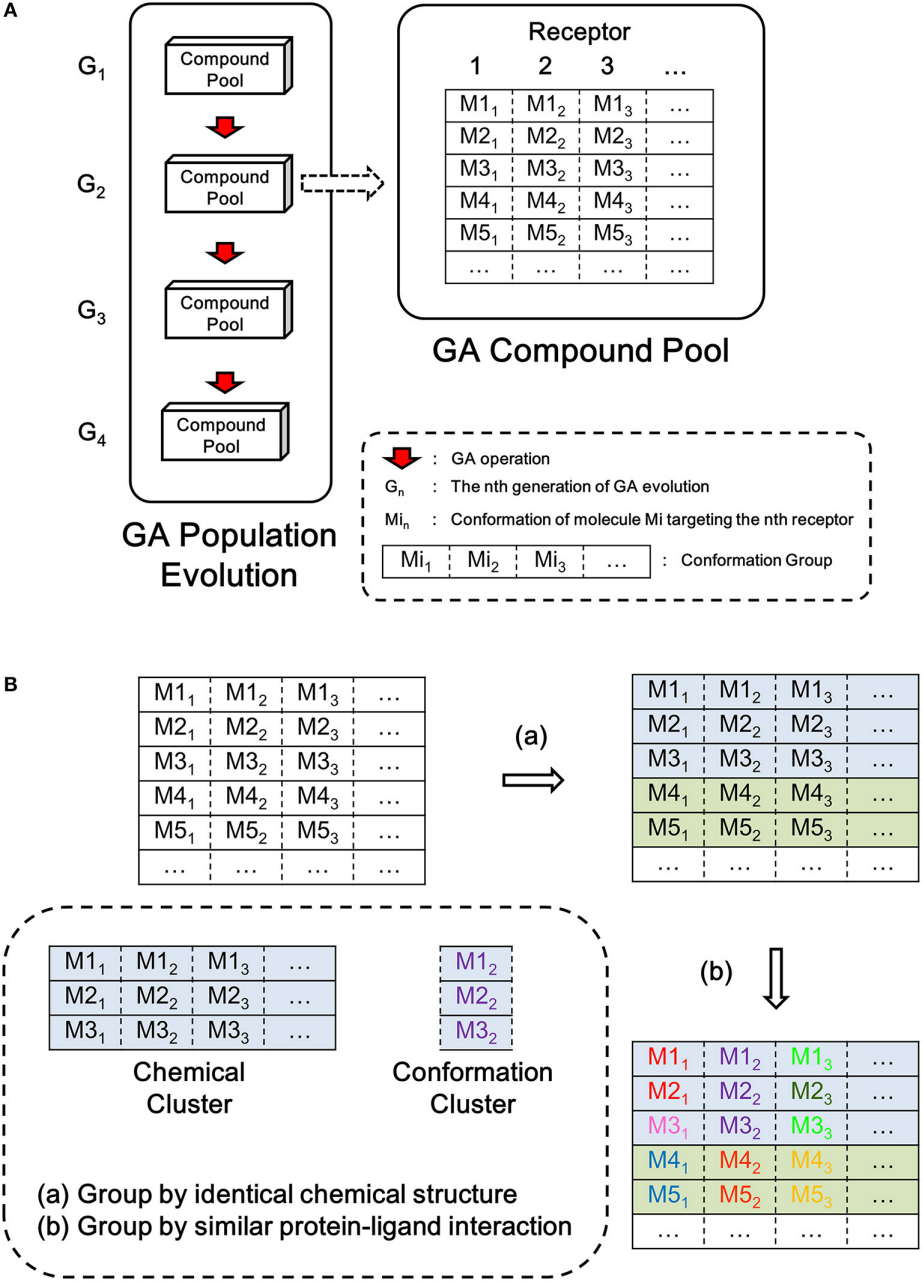
Sketch map of data structure in LigBuilder V3. **(A)** The overview structure of GA evolution and GA compound pool. The GA compound pool includes the ensemble of molecules evolved in GA population. For multi-target design, conformations for each receptor are listed in corresponding column, as a result, each row could represent a solution of multi-target inhibition, and it could be viewed as the basic unit in multi-target GA population, which has similar status as “molecule” in single-target GA population. To avoid confusion, we define each row as a “conformation group” instead of “molecule” or “conformation.” **(B)** The definition of chemical cluster and conformation cluster. The compound pool is clustered on two levels: **(a)** all molecules sharing identical chemical structures will be clustered as chemical cluster, which could be synthesized by same route; **(b)** conformations with similar protein-ligand interaction will be clustered as conformation cluster, which represent same interaction mode.

LigBuilder V3 implements the same genetic algorithm (GA) (Fraser, 1957; Bremermann, 1958; Holland, 1975; Whitley, 1994) used in LigBuilder V2. GA is an optimization algorithm inspired by the process of natural selection, and it mimics the evolution of a population under selection pressure. LigBuilder V3 uses the overlapping generation model of GA, that is, new generation of individuals are evolved from previous population and then replace their parents with GA iteration. For a typical overlapping generation model of GA, roulette wheel selection approach is used to select 10% members from current population as parent for evolving next generation, and all members in current population will be discarded. To balance quality and diversity of population, LigBuilder exempt the top 10% members in current population from elimination, that is, these top members will be directly transferred to next generation. So the quality of member in offspring generation will be better, at least equal to parent generation. We define the GA compound pool as the ensemble of molecules in the newest generation of GA population evolution. The overview of the data structure used in GA evolution is described in Figure 1A.

As each molecule produced by multi-target design method involves multiple proteins, we should consider the multiple conformations of the molecule that bind to its corresponding targets. This is different to single target drug design method. To avoid confusion, we use “Conformation Group” instead of “Molecule” and “Conformation” to indicate the multiple binding conformations of multi-target molecule in this manuscript (Figure 1A).

We also define the “chemical cluster” and “conformation cluster” to depict the relationship in chemical structure and binding conformation among multi-target molecules (Figure 1B). From the perspective of synthesis, molecules with the same chemical structure could be considered as identical. But from the perspective of protein-ligand interaction, the conformations of ligand must be taken into account because the binding of ligand is based on spatial interaction between atoms from ligand and protein. Therefore, we cluster all conformations at two levels: (1) chemical clusters: each conformation of a chemical cluster shares the same two-dimensional (2D) structure, and they could be synthesized via the same reactions estimated by the synthesis-accessibility analysis module inherited from LigBuilder V2; (2) conformation clusters: all the conformations in a conformation cluster also share the same 2D structure, thus the conformation cluster is a subset of chemical cluster. All conformations in a conformation cluster are similar with each other, so they could be consider as sharing same interaction mode. Although all conformations of a conformation cluster are interchangeable from the perspective of interaction mode, we have to keep these “duplicates,” because they may provide necessary local perturbation, for example, the members in a conformation cluster may have various orientations of hydrogen atoms. The orientations of hydrogen atoms usually have little effect on protein-ligand binding except being involved in hydrogen bond forming, but it is much sensitive in further evolution of molecules because the hydrogen atom is responsible for growing site for connecting newly added fragments.

### Multi-Target Seed Structure Mapping

**Figure 2:**
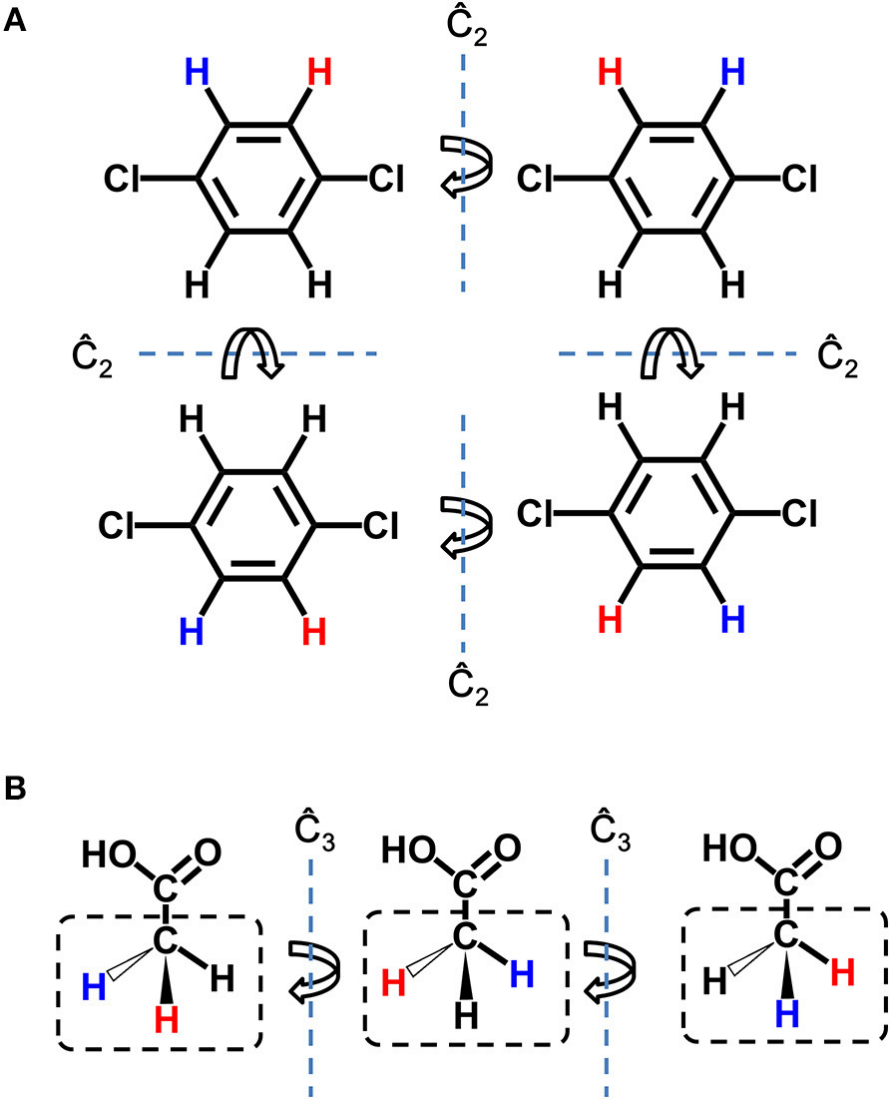
Symmetry in hydrogen mapping. **(A)** Example for the symmetry of structure. 2 C_2_ symmetry axis of 1,4-dichlorobenzene conduce to 4 possible hydrogen mappings. **(B)** Example for the symmetry of group. A C_3_ symmetry axis of methyl group of acetic acid conduces to three possible hydrogen mappings. The hydrogen atom colored in red and blue indicates the first hydrogen and last hydrogen in hydrogen mapping, respectively.

Seed structure is the starting point structure for lead optimization. The preparation of seed structures for single target lead optimization is straightforward, however, additional steps are needed for preparation of seed structures for multi-target design. As each “multi-target seed structure” indicates a conformation group which is composed by the different binding conformation of the ligand to each target, therefore it is necessary to make one-to-one correspondence between atoms of each member in the conformation group. Because only hydrogen atoms are possible connection site in the whole design process, the seed structure mapping is based on the mapping of hydrogen atoms, namely, hydrogen mapping. Due to the symmetry of molecule, there may be more than one possible solution of hydrogen mapping between two structures. As depicted in Figure 2, two types of symmetry should be taken into account, i.e., the hydrogen symmetry of molecule and the hydrogen symmetry of group. The molecular hydrogen symmetry refers to the rotation symmetry of all hydrogen in the molecule, and the hydrogen symmetry of group refers to the rotation symmetry of multiple hydrogen atoms that connected to one heavy atom. Figure 2A shows two C_2_ symmetry axises of 1,4-dichlorobenzene, which conduce to 4 possible hydrogen mappings. Figure 2B shows a C_3_ symmetry axis of the methyl group of acetic acid, which conduces to 3 possible hydrogen mappings. We should note that although some molecules such as the acetic acid are not chiral, the potential chirality is taken into account for the hydrogen mapping in LigBuilder V3, because the further growing operation may bring in chirality to the carbon atom. In other words, both 2D topological and three dimensional (3D) structural information are considered in hydrogen mapping.

Although all the hydrogen atoms in ligand are possible fragment growing site, not every hydrogen atom could serve as growing site because of steric hindrance or user's preference. Thus, LigBuilder V3 only reserves the possible hydrogen mappings with maximal growing sites mapped, named growing site mapping, which increase the possibility of further growing operation to the greatest extent. If there is no possible hydrogen mapping or growing sites mapping, the corresponding conformation group will be ignored. However, if there are more than one solutions of rational hydrogen mappings, LigBuilder V3 will regard them as different seed structures and use them independently in subsequentially design process.

For the case that involves more than two targets, LigBuilder V3 makes hydrogen mapping between the conformation for the first target and each of the remaining targets one by one. As a result, all the rest conformations of the conformation group are mapped to the first conformation, so it is feasible to the find the common growing site mappings of the whole conformation group.

### Multi-Target Growing

Lead optimization is the fundamental function of LigBuilder series. Both LigBuilder V1 and V2 provide the “Growing” strategy, which generates derivatives based on the lead structure (i.e., “seed” structure) that has been pre-placed into the binding pocket. In the present study, we extend the “Growing” strategy to multi-target growing (multi-target lead optimization).

**Figure 3:**
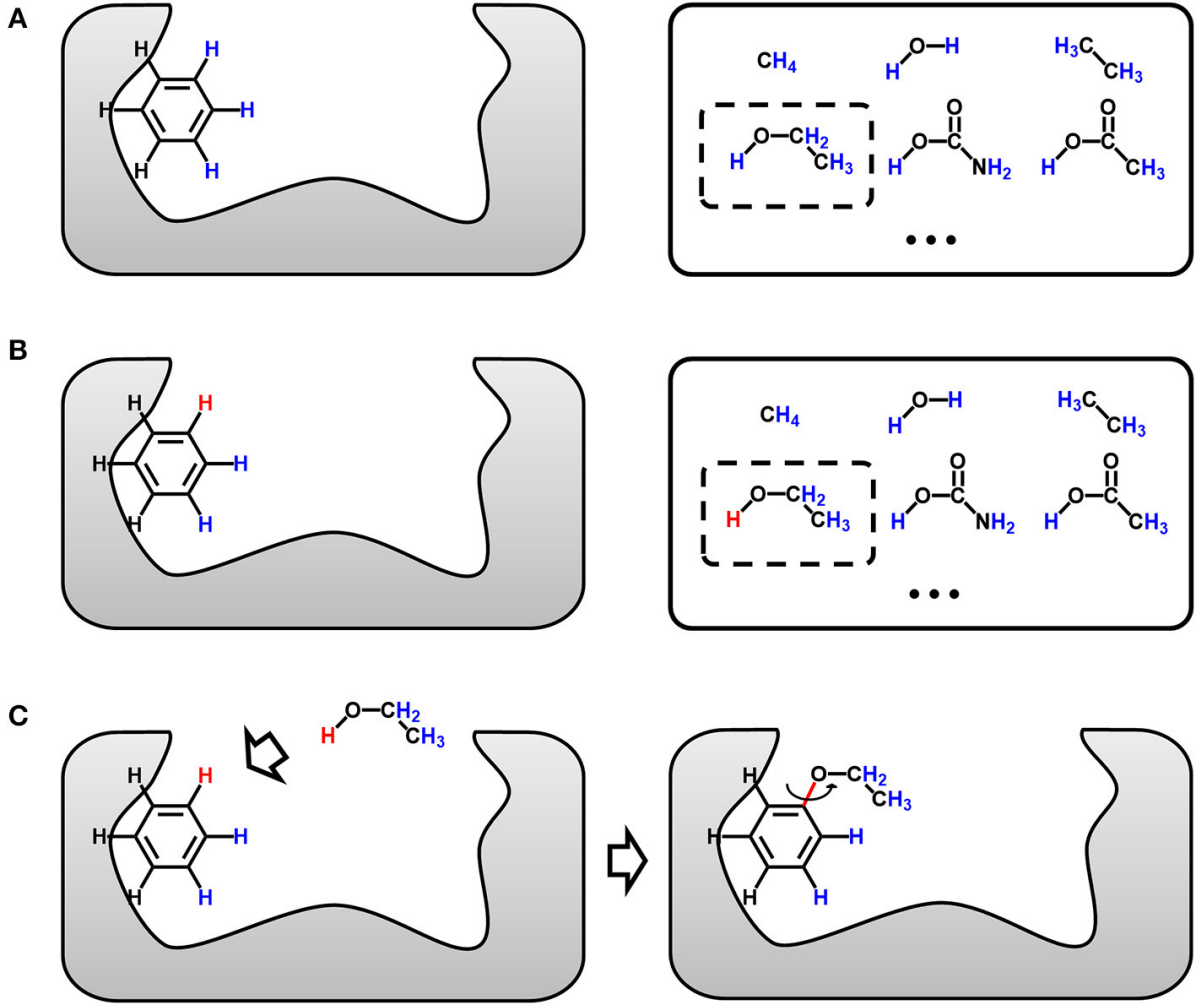
Sketch map of the growing operation. **(A)** The seed structure in the binding site and chosen fragment (dashed box) in the building block library (solid box). All potential growing sites are colored in blue. **(B)** The randomly chosen growing site of seed structure and chosen fragment are colored in red. **(C)** The chosen fragment is moved and then attached to the seed structure. The favorable conformations are determined by uniformly 3 degree-step sampling of the torsion angle along the newly formed bond, which is colored in red.

Figure 3 is the sketch map of single-target growing operation, which is the basis of multi-target growing operation. The gray area on the left in Figure 3A represents the binding site of the target, and the benzene is a representative seed structure. Molecules in the solid box on the right are privileged fragments, which could serve as the building blocks for assembling new structure. Although all hydrogen atoms are feasible for attaching fragments, only a few of them are potential connection site without steric hindrance. Taking Figure 3A for example, hydrogen atoms of benzene face to the vacant region of binding site are colored in blue, which indicate the potential growing sites, and the others near to the receptor atoms will be ignored. Meanwhile, all the hydrogen atoms of building blocks will be considered as potential connection site by default. Users can also assign or block certain “growing sites” on seed structures and building blocks to customize the style of molecule. As the seed structure and building block library has been prepared, LigBuilder will randomly choose a fragment from the building block library (the dashed box in Figure 3A), and then randomly choose a potential growing site on the seed structure and the chosen building block, respectively (red hydrogen atoms in Figure 3B). The building block will be attached to the seed structure along the direction of selected hydrogen atoms (red hydrogen in Figure 3B). With uniformly 3 degree-step sampling of the torsion angle along the newly formed bond (red bond in Figure 3C), several favorable conformations with local minimal energies will be reserved as candidates in consideration of the flexibility of molecule. GA is applied to select elites from these candidates, and these elites will serve as the seed structures for the next growing cycle. This repeated process for each ligand continues until: (1) the ligand is fully designed and there is no available space for adding any new chemical group; (2) the ligand reaches the limitation of molecular weight, which is 480 Da by default; (3) the GA generation number reaches a maximal number, which is 15 by default.

**Figure 4:**
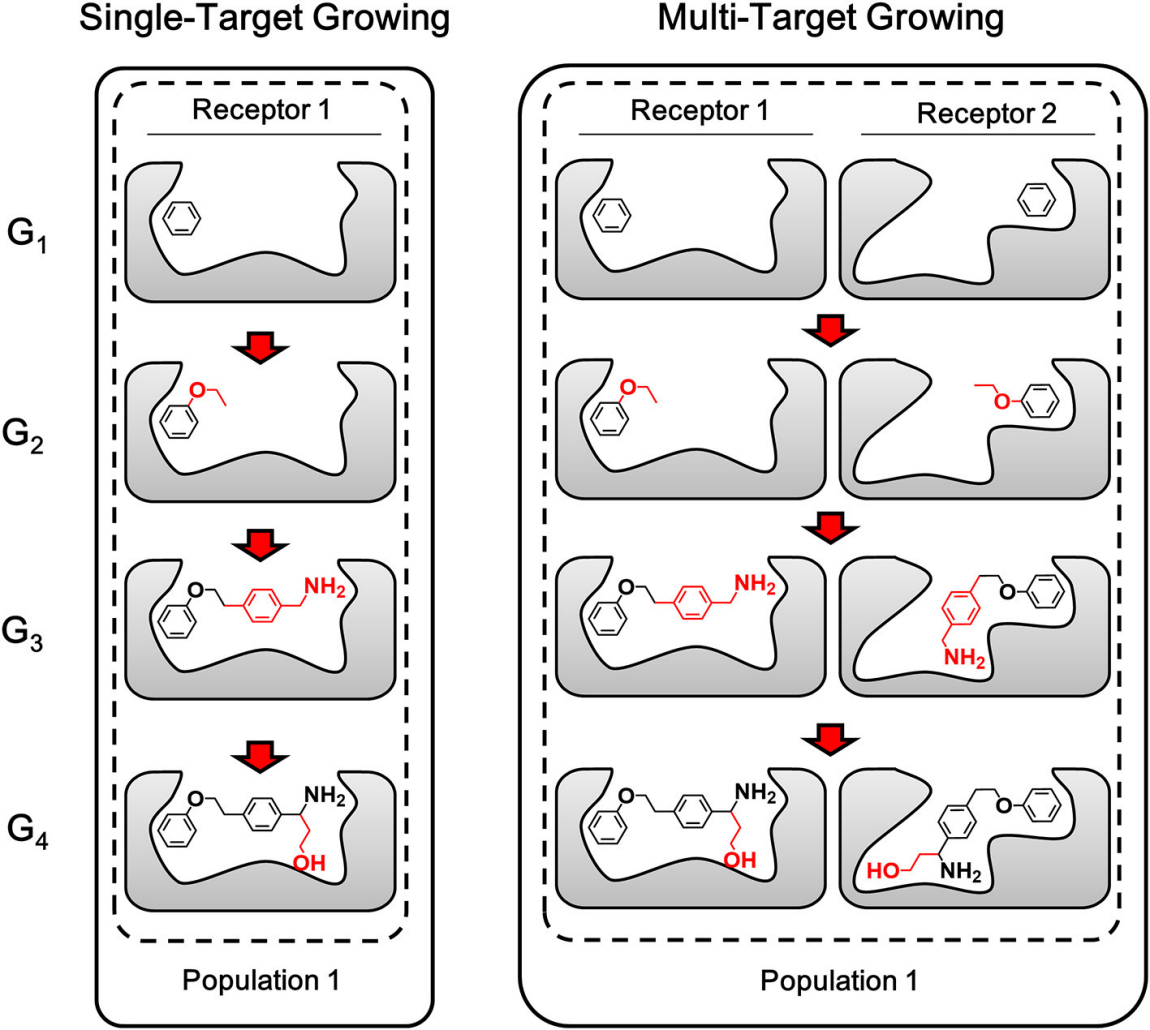
The sketch map of growing process. The multi-target growing operation could be considered as multiple synchronous single-target growing operation. The fragments grown in each step are colored in red (Only one conformation group of each generation are showed here as representatives of the compound pool).

Different from single-target design, the lead structure for multi-target design should be prepared as “seed” conformation group, which is composed by the binding conformation of the lead structure to each target. With a simultaneous operation of growing chemically identical building block on the same site of each member in the conformation group, compounds generated by LigBuilder V3 are expected to be capable of binding to multiple targets. As depicted in Figure 4, multi-target growing could be considered as multiple synchronous single-target growing operation. The identical building block and the same growing site in the growing operation will maintain the consistency of 2D structures of the conformation group. Meanwhile, the 3D conformation of ligand is only restrained by its corresponding targets, that is, the conformation in each conformation group is optimized and evaluated independently. Therefore, this strategy could utilize the flexibility of ligand to improve the capability of multi-target binding. Genetic Algorithm (GA) is also applied to manipulate the growing cycle in the same manner as single target growing.

### Ensemble Linking

Although assembling several bioactive fragments to generate potent ligand is very promising, the computational method of linking proximal fragments covalently is fraught with challenges. To avoid affecting respective bioactivity, the orientation and position of fragments should be changeless. Therefore, the feasibility of linking is severely limited by the rigid restriction of bond length and bond angle in molecule. Besides, the unfavorable energy of torsion may further reduce the feasibility. Although there may be some solutions existed in huge chemical space, the low efficiency in finding these solutions narrows the application of fragments linking. As a result, there are few successful cases of fragments linking, except using flexible chain as linker. However, although flexible chain could be used to relax the rigid restriction of linking, it may increase the amount of accessible conformation of the structure which brings in unfavorable entropy change during binding process, thus the linked fragments usually do not bind as the same degree as the sum of the individual fragments. Moreover, the excessive flexibility of structure may reduce the specificity of ligand.

**Figure 5:**
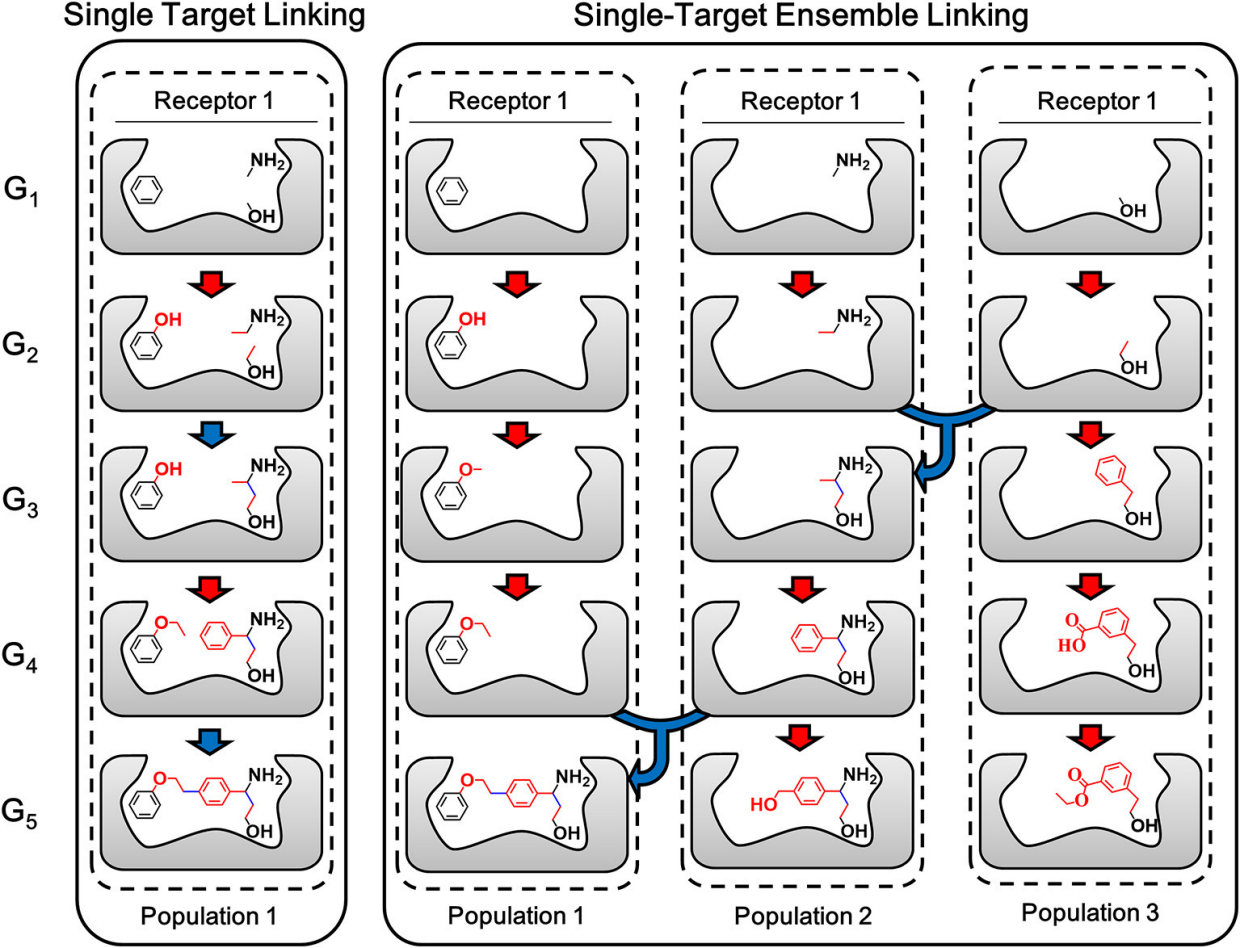
The sketch map of linking and ensemble linking process. The red arrow indicates the growing operation while blue arrow indicates the linking operation. In accordance with the color of arrows, atoms, and bonds formed in growing and linking operation are colored in red and blue, respectively. For previous linking algorithm, the aggregation of certain fragments is regarded as a whole “seed,” so the linking process will be constrained by every fragment in the aggregation. Instead, ensemble linking algorithm applies a more flexible linking strategy, which attempts to derive new structures from each of seed fragments independently and then find possible way of linking among these structures. The different populations indicated different independent GA threads.

The ensemble linking algorithm is developed in LigBuilder V3 to improve the efficiency of fragments linking process, which is expected to make this method more practical. To demonstrate the details of the new algorithm, the sketch map of the linking algorithm used in previous versions of LigBuilder series and ensemble linking algorithm used in LigBuilder V3 are compared in Figure 5. Previous linking algorithm applies a direct strategy of linking, which aims to linking certain fragments with many building blocks. Instead, ensemble linking algorithm applies a more flexible linking strategy, which attempts to derive new structures from each of seed fragments independently and then find possible way of linking among these structures. Although both algorithms are capable of generating the same final structure in Figure 5, ensemble linking strategy is expected to be more efficient. For the general linking algorithm, it is straightforwardly requiring that all the given fragments should be linked, which is usually hard especially for multi-target linking. To overcome this problem, ensemble linking algorithm is based on extra linking fragments, and automatically find the apportioned combination of fragments, which would improve the possibility of finding solution for linking fragments. To be specific, for general linking algorithm, the number of fragments used for linking is limited, for example, user poses 3 fragments into the ligand binding site, and the linking algorithm attempts to find suitable linkers to connect all these 3 specific fragments. For ensemble linking, user could pose several thousands of fragments into the ligand binding site, and the ensemble linking algorithm attempts to find suitable linkers to connect any 3 fragments among all available fragments. Obviously, the ensemble linking algorithm will significantly increase the possibility of finding suitable solution for linking 3 fragments comparing with general linking algorithm. Besides, with dissociation of combined seed fragments in linking algorithm, the whole linking process would be more robust, it would not be dragged by improper derivation or conformation of individual fragment. Moreover, it raises the possibility of comprehensive utilization of more bioactive fragments without exhaustive combination. As a result, LigBuilder V3 could be applied to find possible solutions of linking among hundreds of fragments, which further improves the success rate of linking.

The ensemble linking algorithm will generate many derivative candidates based on the linking fragments to enhance the possibility for finding solution, obviously, although the possibility of linking increases with the number of candidates, the computation cost also will increase by the same rate. So it is important to generate candidates more effective rather than increase the number of candidates to improve the efficiency. As all candidates in the GA population have similar molecular weight because they are generated by GA evolution with same number of generations, the linking possibility will reach the peak when candidates in the population occupy about half of the binding site. But it rapidly falls when candidates in the population are too large to be integrated in limited space of binding site. In addition, the linking possibility is also low when the candidates in the population are too small which may make them far away from each other for linking. So we applied a stagger strategy that operating several independent GA threads simultaneously, meanwhile, the starting of each GA threads are staggered so as to make them be in various generation of GA process. That is, ensemble linking algorithm will not only perform “intra-linking” among candidates in a GA process, but also perform “inter-linking” among candidates in different GA threads. With this strategy, the high diversity of molecular weight distribution among all candidates could bring in higher linking possibility and efficiency.

**Figure 6:**
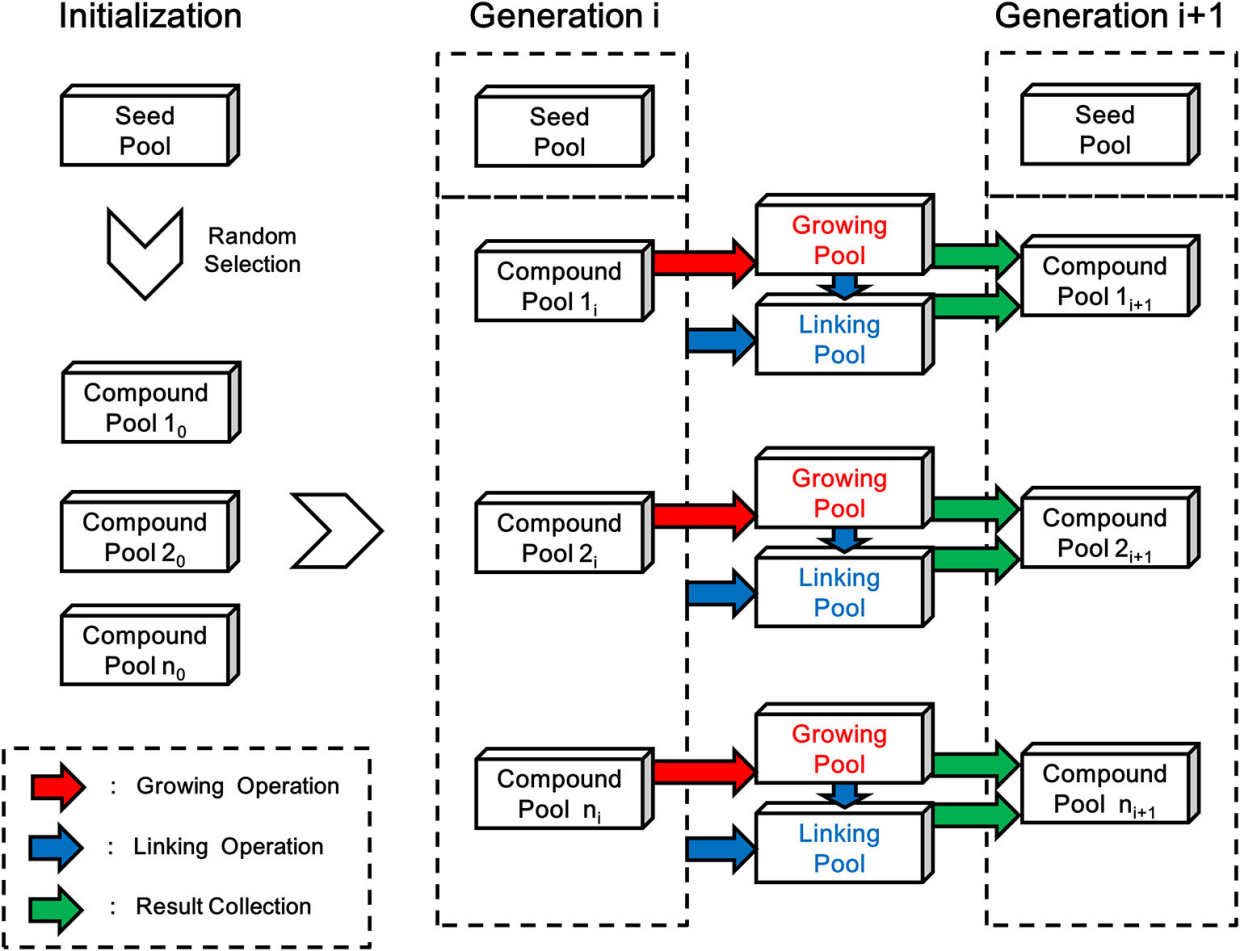
The flow chart of linking process in LigBuilder V3. The structures of initial compound pool are randomly selected from the seed pool in the initialization stage. Then LigBuilder V3 perform the growing and linking operation on each compound pool for producing new generation of compound pools. The growing operation brings in derivation on molecules in previous compound pool and result in a “Growing pool,” which is colored in red. Then the linking operation will be performed for finding possible way of linking between these newly formed structures and previous existed structures. The “previous existed structures” in each generation is denoted with the dashed box. As similar with growing operation, the linking results will be collected into a “Linking pool” which is colored in blue. At last, the “Growing pool” and “Linking pool” will be merged into a new generation of compound pool.

As depicted in Figure 6, each generation of ensemble linking can be decomposed into two steps, i.e., the growing step and the linking step. LigBuilder V3 performs the growing operation on all compounds from each compound pool in the growing step, and then finds possible way of linking between the newly formed compounds and all previous existed compounds including seed pool (dashed box in Figure 6) in the linking step. Although compounds generated in both steps will be collected together into new generation of compound pools, the compounds generated in the linking step (linking pools in Figure 6) will have a certain level of priority in GA process, which make the ensemble linking algorithm trends to link fragments rather than grow for derivation. To be specific, the compounds generated in the linking step indicates a “linking” operation is occurred, on the contrary, compounds generated in the growing step do not link with other fragments in this step. So LigBuilder will elevate the fitness score of compounds from linking step, which encourage the linking behavior. The structures of initial compound pool are randomly selected from the seed pool. After the initialization, LigBuilder V3 will repeat the ensemble linking process for each ligand until: 1) the ligand is fully designed and there is no available space for any new chemical group; 2) the ligand reaches the limitation of molecular weight, which is 480 Da by default; 3) the GA generation number reaches a maximal number, which is 15 by default.

### Multi-Target Linking

A further challenge lies in designing multi-target ligand is linking fragments that interacting with multiple targets. Although many successes occurred in designing single target ligand by fragments linking strategy, few research focus on multi-target linking method. Comparing with lead compound, potential active fragments are much easier to pick by fragment-based approach, such as NMR, DSF, X-ray crystallography, surface plasmon resonance and mass spectrometry (Mashalidis et al., 2013). In addition, computational methods such as fragment docking (Wang et al., 2015) or CrystalDock (Durrant et al., 2011) are also effective ways to identify lead-fragments. Moreover, small fragments are much more likely to interact with multiple targets due to its lower specificity. Therefore, it is feasible and promising to design multi-target ligand by integrating several fragments. So we try to improve our ensemble linking algorithm to handle multi-target fragments linking in LigBuilder V3.

As with the multi-target growing algorithm, the fragments for multi-target linking should also be prepared as “seed” conformation group. Because the ensemble linking algorithm in LigBuilder V3 handle the fragments independently, users no longer have to predetermine which fragments will be linked together in the stage of seed preparation. That is, the procedure of conformation group preparation for linking is same as that for growing. The only difference is that at least 2 conformation groups should be prepared for linking and at least 1 for growing.

**Figure 7:**
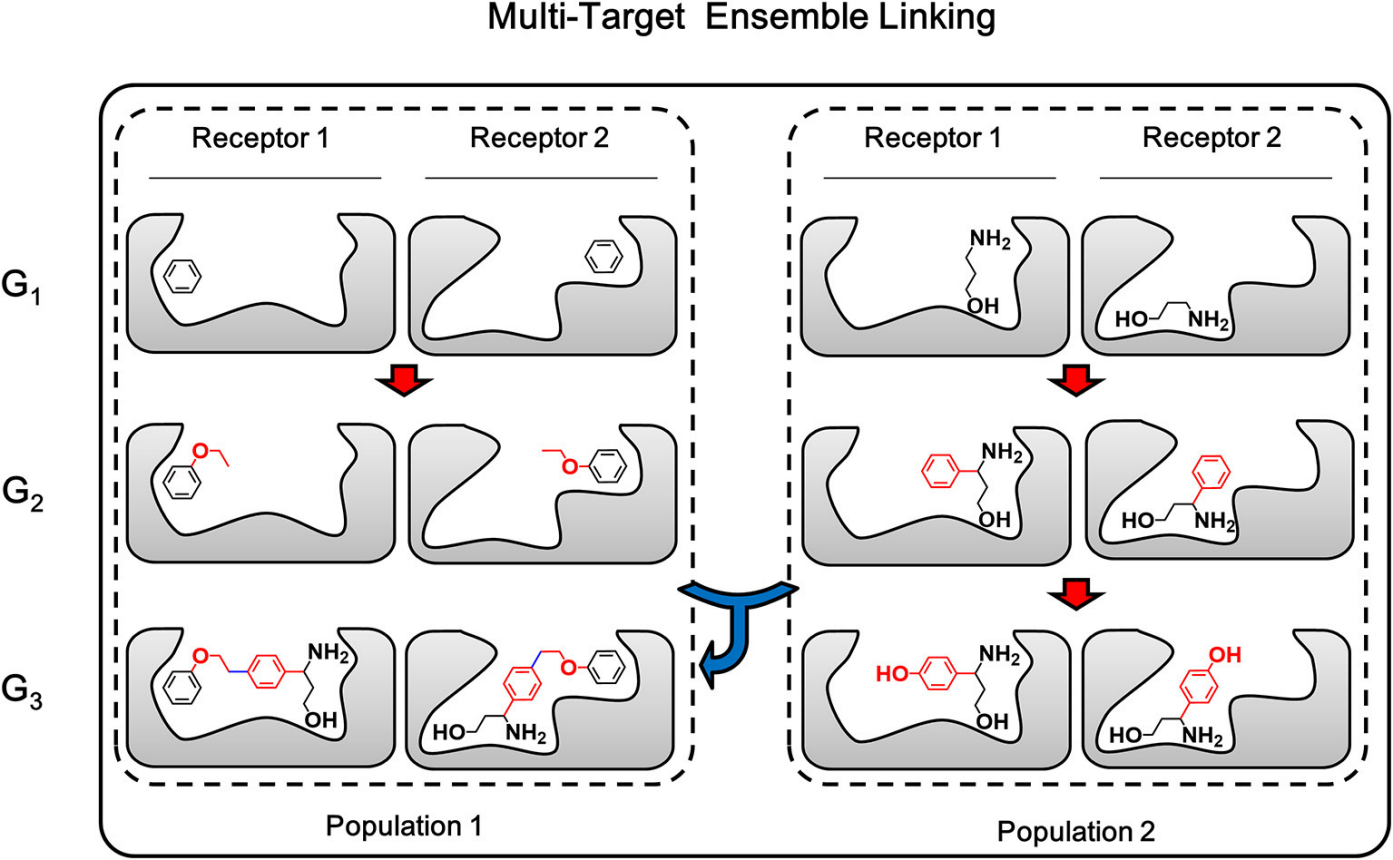
The multi-target ensemble linking operation could be considered as multiple synchronous single-target ensemble linking operation. The red arrow indicates the growing operation while blue arrow indicates the linking operation. In accordance with the color of arrows, atoms, and bonds formed in growing and linking operation are colored in red and blue, respectively.

Multi-target linking algorithm is based-on the ensemble linking algorithm described in above section, therefore both the growing step and linking step which make up the ensemble linking algorithm will be extended to multiple targets. The growing step of multi-target ensemble linking is exactly the same with multi-target growing, while the linking step applies the same strategy of “multi-target operation” used in multi-target growing. As described above, multi-target growing operation is a simultaneously operation of growing chemical identical building block on the same site of each member in the conformation group. In a similar way, multi-target linking operation in linking step is a simultaneously pairwise operation of linking corresponding members from two conformation groups on the same linking sites (Figure 7). That is, the first member of conformation group A will be linked with the first member of conformation group B. Then the second member of both conformation group will be linked together on the same linking sites of the first member. This pairwise process will be repeated until all members have been linked or any failure occurs due to steric hindrance or molecular tension. As a result, these linked structures are expected to be capable of binding to multiple receptor, while they share identical chemical structures.

### Multi-Target *de novo* Design

**Figure 8:**
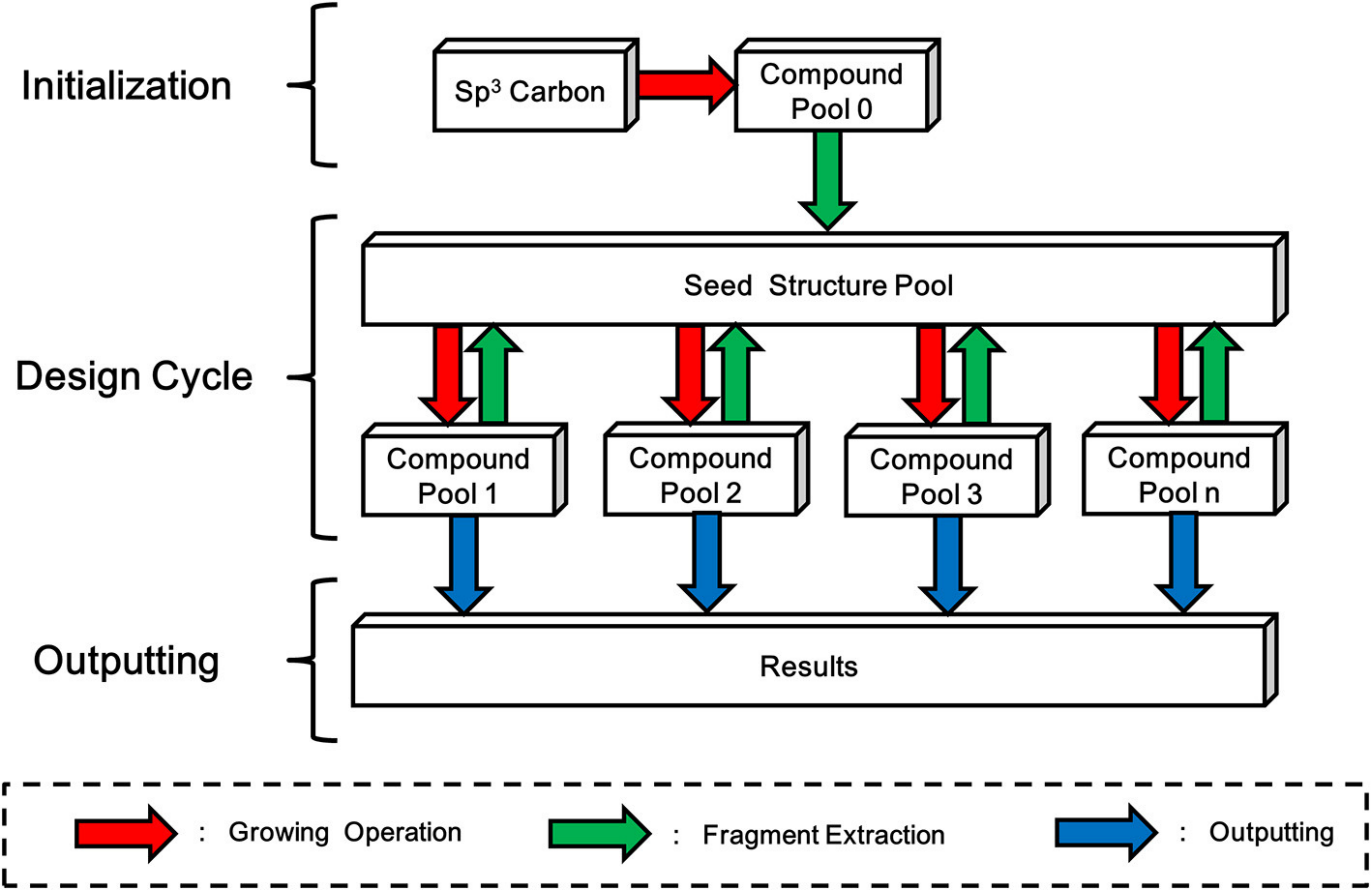
Schema of Chemical Space Exploring Algorithm (CSEA). A randomly positioned sp^3^ carbon will be taken as starting point of growing operation for generating the initial compound pool. Then fragments extracted from compound pool will be used to construct a seed structure pool, which contains seed structures that more favorable than the initial sp^3^ carbon. As new compound pools could be generated based-on structures in seed structure pool, while fragments extracted from these newly formed compounds will be used to update the seed structure pool, LigBuilder V3 avoids the dependency of initial structure and be promising in exploring larger chemical space.

LigBuilder V3 inherits the “Chemical Space Exploring Algorithm” (CSEA) from LigBuilder V2 to create novel scaffolds and structures. In LigBuilder V2, CSEA works in the following way: (1) an sp^3^ carbon with 4 hydrogen atoms will be randomly posed in the binding site and serve as the starting point of constructing new molecules with growing operation; (2) newly designed molecules will be split into fragments; (3) fragments with high predicted binding affinity, that is, high contribution fragments will be selected for updating the “seed structure pool,” which is used to supply seeds for subsequent design cycles; (4) a structure from “seed structure pool” is randomly select as the starting point of constructing new molecules with growing operation, then the 2–4 steps will be repeated. For LigBuilder V3, CSEA is extended to multi-target design purpose by applying multi-target growing operation instead of single-target growing operation. Meanwhile, the fragment extraction process of single conformation will also be replaced by fragment extraction of “conformation group” (Figure 8). With the self-circulation seed generating feature, CSEA can help to avoid the limitations associated with pre-assigned seed structures and explore a broader chemical space, thus greatly improving the novelty and efficiency of design.

As the seed structure pool is used to collect and provide initial fragments of design process, the quality of seed structure pool may significantly affect the design results. Since CSEA could provide a mass of potential seed fragments during the design process, the seed structure pool will be updated to achieve higher binding affinity while maintain diversity of seeds. Then the CSEA will have a higher starting point for generating potent structures, which in turn produce better seed fragments for updating the seed structure pool. Therefore, the seed structure pool will keep evolving during the whole design process, which iteratively optimize the performance of design.

### Seed Structure Extraction

The most direct way of extracting component fragments from chemical structure is splitting the molecule by iterating over all single bonds. However, the traversal extraction method would take a lot of computing time when handle millions of compounds, which is a common order of magnitude in CESA process of LigBuilder V3. Therefore, we develop a simplified extraction algorithm for acceleration. To balance the representativeness of fragments and extraction speed, we only focus on the molecular scaffold and key interaction group, which are major determinants of molecular conformation and protein-ligand interaction. As a result, only five categories of fragments are considered in CESA: (1) Single atoms; (2) rigid scaffold (rigid chain and rigid ring system); (3) flexible scaffold (flexible ring system); (4) interaction group (e.g., carboxyl group); (5) scaffold with connected interaction group. If a fragment could be classified into more than one category, it will be put into the category with smallest category number. With this algorithm, LigBuilder V3 is also capable of extracting fragments from known ligands as seed structures, which is convenient for fragments linking or lead optimization.

For multi-target design, as the seed structure is composed of multiple conformations (conformation group) instead of single conformation, seed structures extracted from known ligands of each targets should be paired to construct conformation groups first. LigBuilder V3 extracts fragments from all known ligands in the same way of extracting fragments in single-target design, and then hydrogen mapping algorithm will be implemented among these fragments to find all possible combinations. That is, if a fragment is present in ligand of every targets, it is a common fragment and LigBuilder will mapping this fragment to construct multi-target fragments group as seed structures. However, if this fragment is absent in ligands for any one of the targets, it is not common fragment and the fragment will be discarded.

### Multi-Target Ligand Efficiency

Ligand efficiency (LE) is frequently used to prioritize hits from HTS, and it can be regarded as a guide for selecting efficient fragments for further optimization. It is much more important for multi-target design because the ligand efficiency is also the index of “integration degree,” which is a more critical index for multi-target drugs. There are various definitions of LE but most widely used approximation is described as the average free energy of binding per heavy atom or average pIC_50_ per heavy atom, which are demonstrated as follows (Hopkins et al., 2004):

$$\begin{array}{l} LE=\frac{-\Delta G}{HAC}\;or\;\\ LE=\frac{-\log\left(IC_{50}\right)}{HAC}\;\left(HAC\;is\;heavy\;atom\;count\right) \end{array}$$

For multi-target ligand, multi-target ligand efficiency (MLE) could be derived with a similar form of LE. As the multi-target ligand causes multiple binding free energy, MLE could be described as the summation of average free energy of binding per heavy atom or summation of average pIC_50_ per heavy atom, which are demonstrated as follows:

$$\begin{array}{l} MLE_{N}=\frac{\sum {-\Delta G}_{n}}{HAC}\;or\;\\ MLE_{N}=\;\frac{\sum -\log\left(IC_{50_{n}}\right)}{HAC}\;\left(N\;is\;the\;number\;of\;target\right) \end{array}$$

The ligand efficiency is not comparable if the target number is different, so we use the subscript for MLE to indicate the condition of rational comparison and make it distinct from LE of single-target ligands. We should note that the MLE is insufficient for the performance evaluation of multi-target ligand, because the uneven activity of targeting individual binding site may be obscured by summation. However, it is much complicated to evaluate the efficiency of multi-target ligand, because it depends on the specific biological network that it is involved in. So the MLE would be only considered as an index of average efficiency for selecting potential multi-target lead structures.

### Other Functional Modules

The other functional modules implemented in LigBuilder V3 are directly inherited from LigBuilder V2, including: (1) drug-like and privileged building blocks; (2) Toxic fragments; (3) Drug-like rules (for example, Lipinski rule); (4) Ligand-binding site detection module; (5) Synthesis analysis modules; (6) Scoring function; (7) LogP module; (8) GA fitness function (composed of scoring function, MLE, toxic fragments filter, and drug-like rules). It should be noted that the binding affinity predicted by scoring function for multi-target ligand is calculated by its average of binding affinity predicted for each target.

### Design HIV-PR/HIV-RT Dual-Functional Inhibitor

#### Structural Preparation

The crystal structures of PR and RT used in this study are downloaded from the RCSB Protein Data Bank (Berman et al., 2003) (PDB code: 3A2O, Hidaka et al., 2009, and 4G1Q, Kuroda et al., 2013, respectively), and both structures are complexes with potent inhibitors solved at high resolution (0.88 and 1.51 Å, respectively). The inhibitor binding sites of PR and RT were defined by binding site detection program Cavity (Yuan et al., 2013; Zhang et al., 2015; Xu et al., 2018), which provides the detailed definition for boundary of “design space.” The drug-like and privileged building blocks used in this study were inherited from LigBuilder V2. Then three different design strategies were used to design dual-function inhibitors for PR and RT.

#### *De novo* Design Approach

LigBuilder V3 inherited the seed generation and optimization algorithm from LigBuilder V2, that is, LigBuilder V3 could iteratively extract seed structures from designed compounds and use the extract seed structures for design new compounds. The GA parameters were set as follows: GA population size of 1,000, GA parent ratio of 10%, GA generation number of 12. Total 1 million candidate dual-functional compounds were generated by LigBuilder V3 with *de novo* design mode.

#### Growing Approach

Growing approach is for optimization of prepared seed structures. We collected all protein-ligand binding complex of PR or RT from the RCSB Protein Data Bank (Berman et al., 2003), including 323 PR-ligand complexes and 141 RT-ligand complexes (Listed in Table S1). All the PR-ligand complexes were aligned to the PR structure complexed with KNI-1689 (PDB code: 3A2O, Hidaka et al., 2009), and all the RT-ligand complexes were aligned to the RT structure complexed with Rilpivirine (PDB code: 4G1Q, Kuroda et al., 2013) using Pymol (Schrodinger, 2010), which could ensure that all ligands in the complexes are also aligned according to the receptor alignment. With the fragment-extraction function of LigBuilder V3, fragments with no more than 20 heavy atoms were extract from these known PR or RT ligands. As the ligand efficiency is important for the seed structure, fragments with SLE index <0.1 were removed, and a total of 2,386 fragments for PR and 1,442 fragments for RT were obtained at this stage. Then fragments for PR and RT with the same 2D structure were paired with “hydrogen mapping” algorithm mentioned above, and a total of 3,506 paired fragments were prepared. The GA parameters were set as follows: GA population size of 1,000, GA parent ratio of 10%, GA generation number of 12. Total 100 K candidate dual-functional compounds were generated by LigBuilder V3 with growing design mode based on the prepared fragments. As the binding affinity is usually related to the size of molecule, large seed fragments are more competitive than small fragments especially for the genetic algorithm used in LigBuilder. So, each fragment was independently used as the seed structure with multiple runs of LigBuilder to avoid bias to large seed fragments.

#### Linking Approach

Linking approach is for integrating key fragments into new compounds. The paired fragments used in this approach were prepared in the same way of growing approach. However, all paired fragments were used together in linking approach to maximize the possibility of finding ways for linking fragments. It would be intuitive that the more fragments provided, the better performance would be expected. The GA parameters were set as follows: GA population size of 10,000, GA parent ratio of 10%, ensemble population number of 10, GA generation number of 12. Total 10 K candidate dual-functional compounds were generated by LigBuilder V3 with linking design mode based on the 3,506 prepared fragments.

#### Post-processing

As multi-target ligand should bind to different proteins with the same chemical structure, ideally, each moiety of the ligand could contribute to its binding to all targets, so ligand efficiency would be important in evaluating a multi-target ligand. In this study, predicted pKd of all the output compounds are larger than 5.0, so only MSLE index were used to rank and select top 1,000 results with best ligand efficiency from multi-target design procedure for the three approaches. Because LigBuilder V3 only uses a fast empirical scoring function for estimating protein ligand binding affinity, in order to improve the accuracy of calculation, the total 3,000 selected compounds were further subjected to energy minimization and 100 ps short time molecular dynamic (MD) simulation by using the Amber package (Case et al., 2012) for estimating the binding affinity with MM/GBSA method (Rastelli et al., 2010).

### Application of LigBuiler V3 in Multi-Target Ligand Design

The concept prototype for growing mode algorithm of LigBuilder V3 has been experimental validated by designing COX2/LTA_4_H dual-functional inhibitor, which resulted in a single ligand that binding to COX2 and LTA_4_H with IC_50_ of 7.1 and 7.0 μM, respectively (Shang et al., 2014). Although this work is based on a developing version of LigBuilder V3, and many manual interventions were involved due to immature of the algorithm, the success of this case suggests the feasibility of using LigBuilder V3 to design multi-target ligand. Moreover, LigBuilder V3 were further developed based on the knowledge learned from this case. Besides the improvement of multi-target growing algorithm, both multi-target *de novo* design approach and multi-target linking approach are realized in this version of LigBuilder V3. In this study, we have tested the LigBuilder V3 by designing dual-functional inhibitor targeting two well-characterized virus enzymes, HIV protease (PR) and HIV reverse transcriptase (RT) with all three design modes. As both PR and RT are important drug targets of clinical antiretroviral therapy, the multi-target strategy such as combination of nucleoside reverse transcriptase inhibitors (NRTI) and protease inhibitor (PI) shows significant advantage over each single component and has been broadly used for HIV treatment (Lu et al., 2018). Consequently, researchers have been interesting in developing cocktail drug combinations, and pursue multi-target anti-HIV inhibitors for improving patient compliance. Matsumoto *et al*. have reported the strategy of linking PR and RT inhibitor by spontaneously cleavable linker (Matsumoto et al., 2000). Furthermore, scaffold merging strategy is successfully applied in designing multi-target anti-HIV inhibitors in recent years (Song et al., 2015; Sun et al., 2016). However, both the dependency of known inhibitors and specific requirement of molecular structure limit the practical applications of structure merging strategy. Therefore, we present a more universal solution of multi-target design with the example of designing dual-functional inhibitors for PR and RT by LigBuilder V3. The detailed methods and parameters are described in the Method and Algorithm section.

The top 1,000 compounds from each design modes were selected and subjected to 100 ps short time molecular dynamic simulation, then the binding affinity of each compounds were estimated by MM/GBSA method. The average binding affinity of the top 10 compounds and top 1 compound for each design modes were collected in Table 1. Although the designed multi-target compounds could not compare with the super potent PR and RT inhibitors with sub-nanomolar level activity, the designed compounds are predicted to be more potent than micromolar level inhibitor of both PR and RT, that is, these compounds are expected to be dual-functional inhibitor for PR and RT at sub-micromolar level activity for both targets.

**Table 1:** Binding free energy predicted by MM/GBSA method.

	***De novo*** **design**		**Growing approach**		**Linking approach**		**HIV-PR potent inhibitor^**b**^**	**HIV-PR weak inhibitor^**c**^**	**HIV-RT potent inhibitor^**d**^**	**HIV-RT weak inhibitor^**e**^**
	**Top 10^**a**^**	**Top 1**	**Top 10**	**Top 1**	**Top 10**	**Top 1**				
PR	−28.2	−35	−28.4	−32	−34.9	−35.0	−68.2	−21.9	–	–
RT	−33.6	−34.9	−34.5	−40.3	−38.0	−38.8	–	–	−41.5	−22.6
Ave.	−30.9	−35.0	−31.5	−36.2	–**36.5**	–**36.9**	–	–	–	–

Energy unit is kcal/mol.

a Top 10 indicates the average of best 10 compounds;

b FDA approved drug Darunavir with IC_50_ of 0.15 nM (Shen et al., 2013). Complex structure was from PDB code 4LL3 (KoŽíšek et al., 2014).

c PR weak inhibitor with IC_50_ of 2.3 μM (Jhoti et al., 1994). Complex structure was from PDB code 1HTE (Jhoti et al., 1994).

d FDA approved drug Efavirenz with IC_50_ of 41 nM (King et al., 2002). Complex structure was from PDB code 1FK9 (Ren et al., 2000).

e *RT weak inhibitor with IC_50_ of 1.2 μM (Chan et al., 2017). Complex structure was from PDB code 5VQS (Chan et al., 2017). Bold values indicate the best average energy among results from three approaches*.

**Figure 9:**
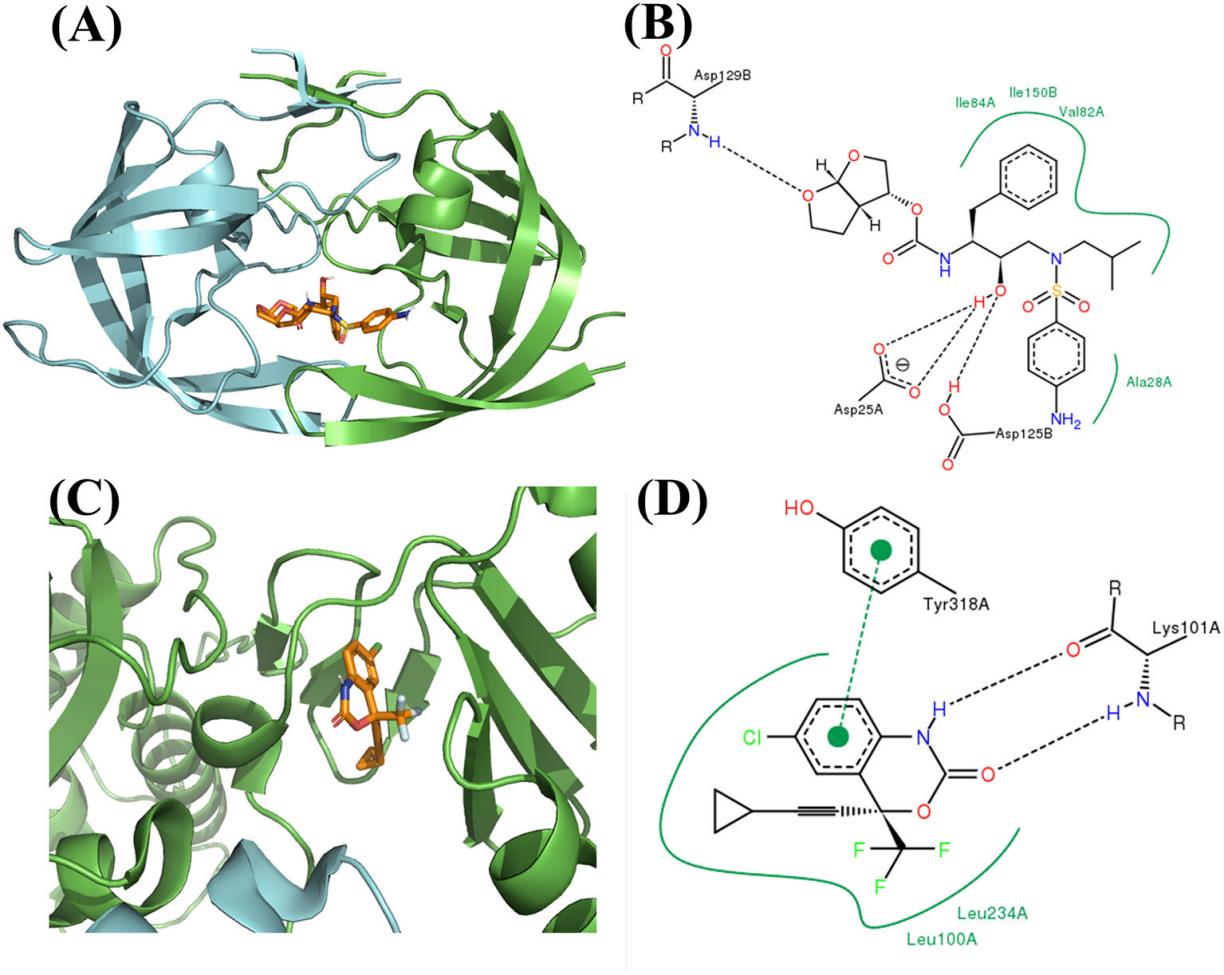
**(A)** Binding mode of FDA approved PR inhibitor Darunavir. The dimerized PR is showed in green and cyan cartoon style, and the Darunavir is showed in golden stick style (figure generated by Pymol, Schrodinger, 2010). **(B)** The 2D interaction figure for Darunavir binding with PR (figure generated by PoseView, Stierand and Rarey, 2010). **(C)** Binding mode of FDA approved RT inhibitor Efavirenz. **(D)** The 2D interaction figure for Efavirenz binding with RT.

**Figure 10:**
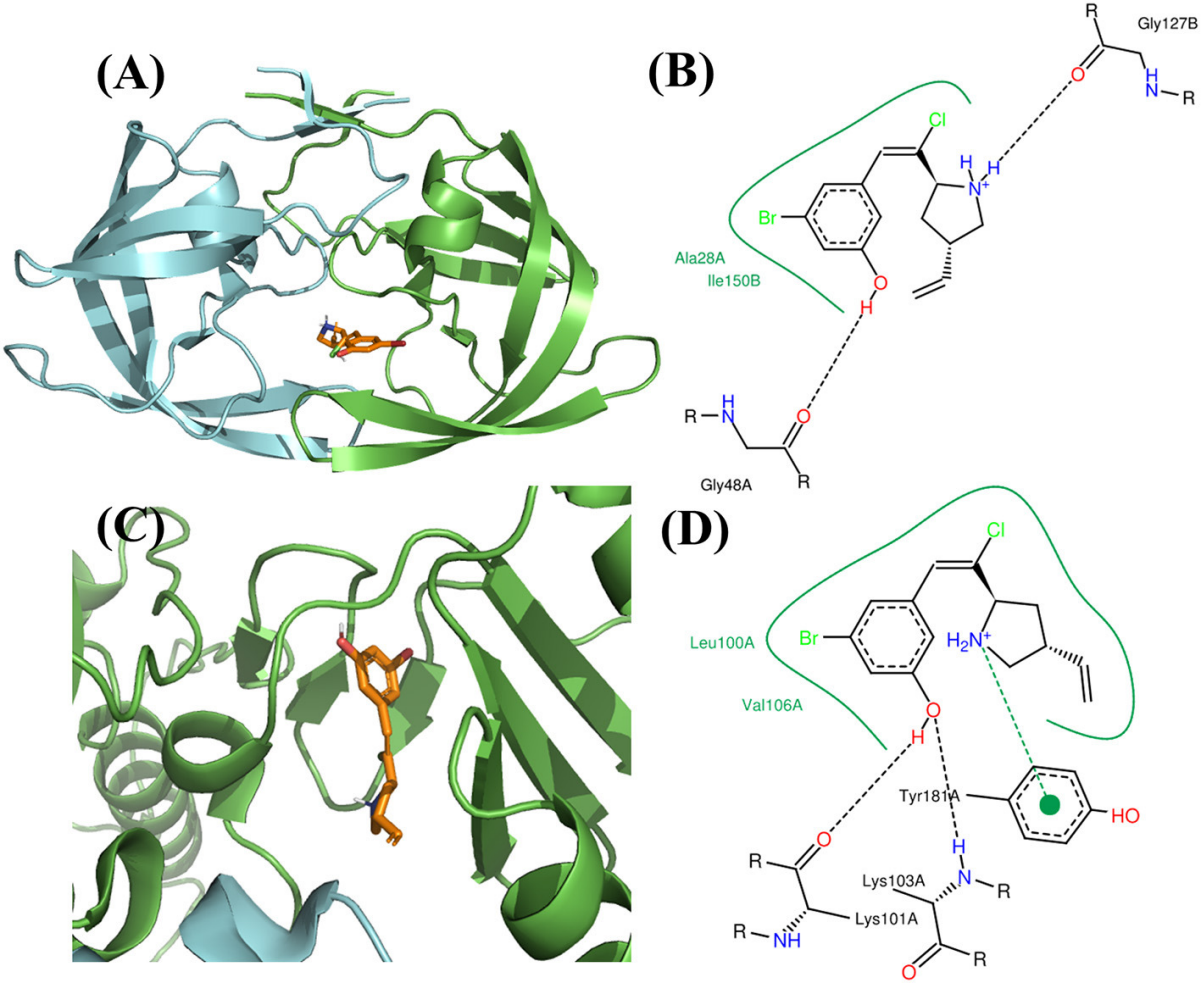
Binding mode of the best compound from *de novo* design approach. **(A)** Binding mode with PR. **(B)** 2D interaction with PR. **(C)** Binding mode with RT. **(D)** 2D interaction with RT.

**Figure 11:**
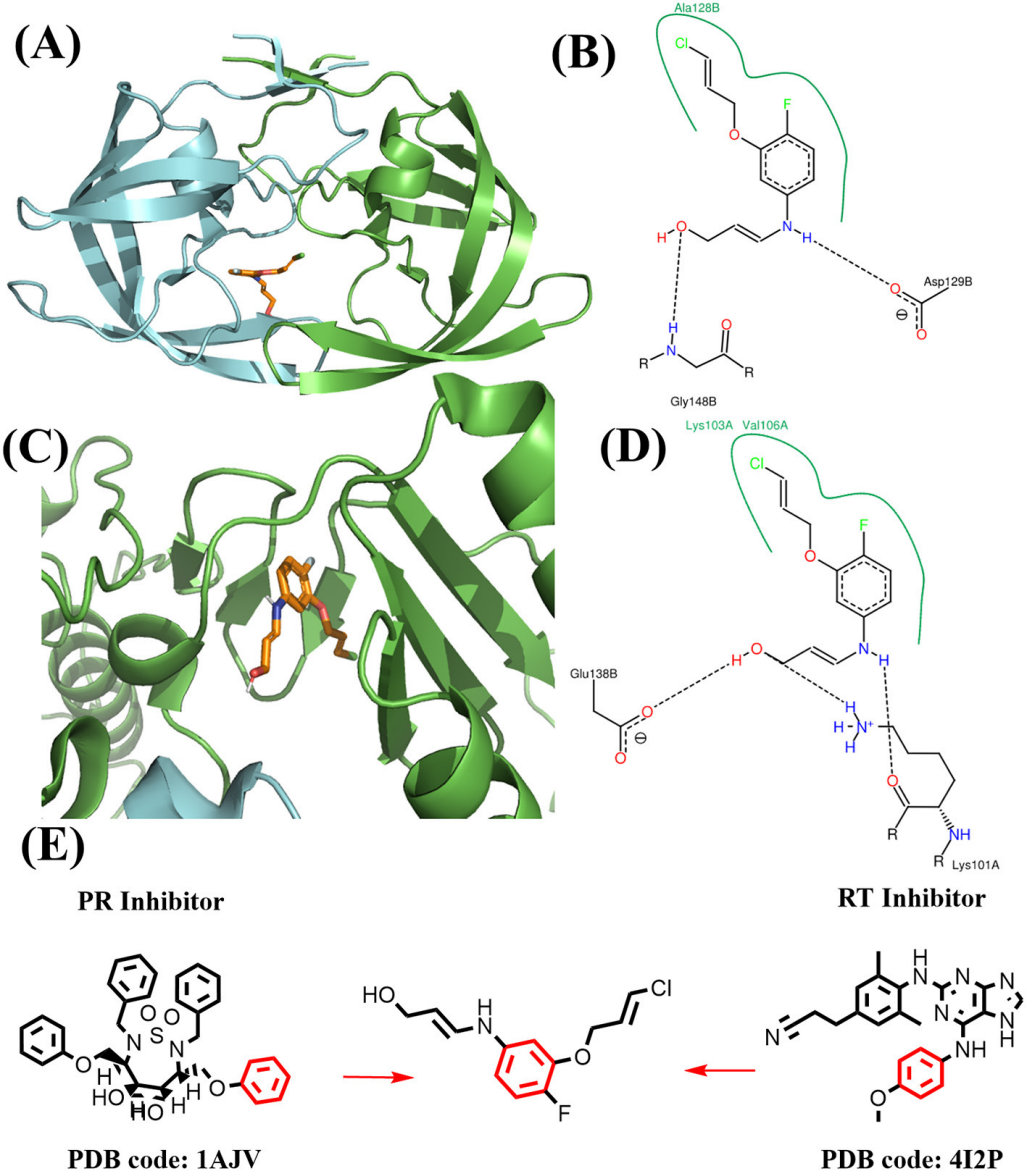
Binding mode of the best compound from growing approach. **(A)** Binding mode with PR. **(B)** 2D interaction with PR. **(C)** Binding mode with RT. **(D)** 2D interaction with RT. **(E)** Source of fragments in PR inhibitor and RT inhibitor for growing are colored in red.

**Figure 12:**
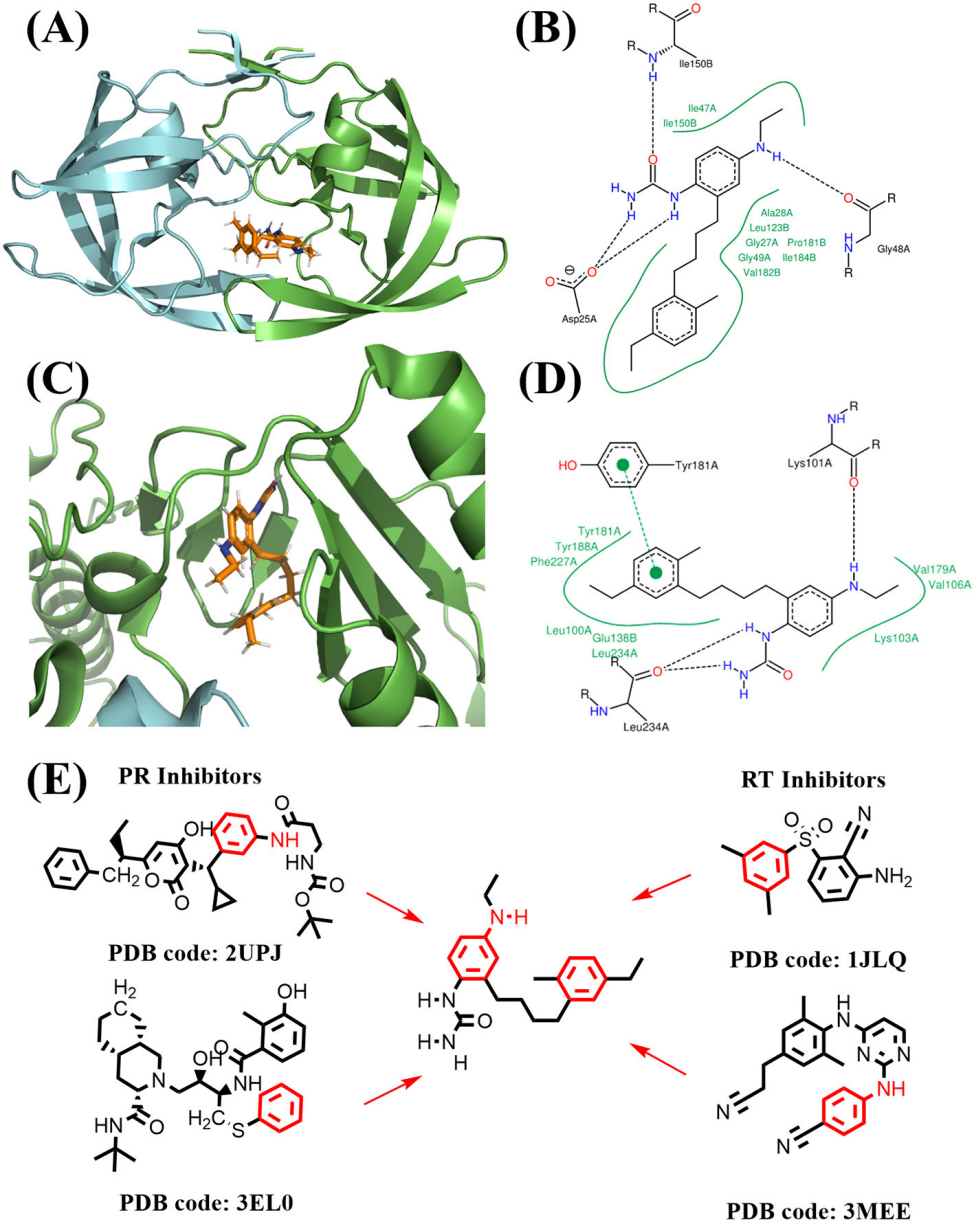
Binding mode of the best compound from linking approach. **(A)** Binding mode with PR. **(B)** 2D interaction with PR. **(C)** Binding mode with RT. **(D)** 2D interaction with RT. **(E)** Source of fragments in PR inhibitor and RT inhibitor for linking are colored in red.

The binding mode of FDA approved PR inhibitor and RT inhibitor are depicted in Figure 9. Obviously, they adopt very distinct protein-ligand interaction modes. The best compound from *de novo* design approach is depicted in Figure 10. This compound forms distinct interaction comparing with known PR or RT inhibitors. It is highly compact and fully utilized its polar groups and hydrophobic groups to form interaction with PR and RT in different manner. The best compound from fragments growing approach is depicted in Figure 11. This compound is growing from a benzene ring which is one of the most common fragments in PR and RT inhibitors. The best compound from fragments linking approach is depicted in Figure 12. This compound is much bigger than compounds from *de novo* approach and growing approach, which indicates its relatively lower ligand efficiency. As linking approach is intensively pursing possible ways for linking provided fragments, the success of linking is more important than ligand efficiency, so the algorithm is preferable to allow generating derivates with much lower ligand efficiency which may enhance the possibility of linking. Overall, all of these compounds are relatively small, and groups in these compounds usually contribute to the binding with different protein in different manner, which is the most need feature for designing highly compact multi-target ligand.

Essentially, the design process of LigBuilder is a kind of “random evolution” process implemented by genetic algorithm. So the quality of design result is expected to be improved along with the total computational time. As the output result could be unlimited, it is not realistic to achieve the maximal quality. For a fair comparison among the results from three strategies, we used different output number to ensure they consumed roughly similar computational time. So we designed 1 million, 100 K, and 10 K compounds for *de novo* approach, growing approach, and linking approach, respectively, which is roughly corresponding to the compound generating efficiency of three approaches in this project. Base on the data in Table 1, linking approach is most effective way of design high affinity ligands, and growing approach is also more effective than *de novo* approach. This is not surprising because known fragments would provide good starting points for derivation and significantly reduce searching space. The linking approach uses more known fragments which further improve its efficiency comparing with growing approach using only 1 fragment. However, the results from *de novo* approach demonstrated that this approach could achieve similar design performance to growing approach or linking approach if more computational resource is provided. As the *de novo* approach does not reply on known fragments, it would be very useful for design ligands for new targets or discover novel ligands for known targets. On the other hand, growing approach and linking approach also have their unique advantages comparing with *de novo* approach. Since compounds designed by growing or linking approach contain “validated active fragments,” it would reduce the risk of “false positive,” which is very common in computer-aided drug discovery. So the three strategies could be complementary in practical drug discovery projects.

## Conclusion

In this paper, we present the first *de novo* multi-target drug design program LigBuilder V3. In addition, building ligands from scratch, LigBuilder V3 also provides the feasibility of multi-target lead optimization and multi-target fragments linking. This program is generally applicable in rational and elegant multi-target drug design and optimization, especially for the design of concise ligands for proteins targets with large difference in binding sites. The developing version of LigBuilder V3 was successfully applied in designing COX2/LTA_4_H dual-functional inhibitors with micromolar level activity. In this study, we further demonstrated the three design strategies of LigBuilder V3 with computational evaluation of designing HIV-PR and HIV-RT dual functional inhibitors. We hope the concept and LigBuilder V3 can be validated by applications from the users in the future.

## Data Availability Statement

All datasets generated for this study are included in the article/Supplementary Material.

## Author Contributions

JP and LL conceived the project. YY, JP, and LL designed the experiments, analyzed the results, and wrote the manuscript. YY performed the experiments.

### Conflict of Interest

The authors declare that the research was conducted in the absence of any commercial or financial relationships that could be construed as a potential conflict of interest.
