# Supplementary material for: LigBuilder V3: A Multi-Target de novo Drug Design Approach
Source: Front Chem. 2020 Feb 28;8:142. doi: 10.3389/fchem.2020.00142 (PMC7059350; doi:10.3389/fchem.2020.00142)
Supplement: Supplementary file 1 [file Table_1.docx]

LigBuilder V3: A Multi-Target *De novo* Drug Design Approach

Yaxia Yuan^1^, Jianfeng Pei^1,2,^*, and Luhua Lai^1,2,3,^*

^1^ State Key Laboratory for Structural Chemistry of Unstable and Stable Species, Beijing National Laboratory for Molecular Sciences, College of Chemistry and Molecular Engineering, Peking University, Beijing, China

^2^ Center for QuantitativeBiology, Academy for Advanced Interdisciplinary Studies, Peking University, Beijing, China

^3^ Center for Life Sciences, Academy for Advanced Interdisciplinary Studies, Peking University, Beijing, China

*Correspondence to: J.-F. Pei, e-mail: jfpei@pku.edu.cn, and L.-H. Lai, e-mail: lhlai@pku.edu.cn

**Table S1. HIV PR and HIV RT complexes used for extracting fragments**

PR complexes:

| 1A30 | 1EC1 | 1KJ7 | 1WBK | 2BPW | 2NXD | 2QNQ | 3EL9 | 3MXD | 3O9G | 3R4B | 3VFA |
| --- | --- | --- | --- | --- | --- | --- | --- | --- | --- | --- | --- |
| 1A8G | 1EC2 | 1KJF | 1WBM | 2BPX | 2NXL | 2R38 | 3EM3 | 3MXE | 3O9H | 3S43 | 3VFB |
| 1A9M | 1EC3 | 1KJG | 1XL2 | 2BPY | 2NXM | 2R3T | 3EM4 | 3N3I | 3O9I | 3S53 | 4A4Q |
| 1AJV | 1F7A | 1KJH | 1XL5 | 2BPZ | 2O40 | 2R3W | 3EM6 | 3NDT | 3OK9 | 3S54 | 4A6B |
| 1AJX | 1FB7 | 1LZQ | 1Z8C | 2BQV | 2O4K | 2R43 | 3FSM | 3NDU | 3OTS | 3S56 | 4A6C |
| 1B6J | 1FEJ | 1M0B | 1ZBG | 2CEJ | 2O4L | 2UPJ | 3FX5 | 3NDW | 3OTY | 3SA3 | 4DFG |
| 1B6K | 1FF0 | 1MT7 | 1ZJ7 | 2CEM | 2O4N | 2UXZ | 3GI0 | 3NDX | 3OU1 | 3SA4 | 4DJO |
| 1B6L | 1FFF | 1MT8 | 1ZLF | 2CEN | 2O4P | 2UY0 | 3GI4 | 3NLS | 3OU3 | 3SA5 | 4DJP |
| 1B6M | 1FFI | 1MT9 | 1ZPK | 2F3K | 2O4S | 2WHH | 3GI5 | 3NU3 | 3OU4 | 3SA6 | 4DJQ |
| 1B6P | 1FG6 | 1MTB | 1ZSF | 2F80 | 2PK5 | 2WKZ | 3GI6 | 3NU4 | 3OUA | 3SA7 | 4DJR |
| 1BV7 | 1FG8 | 1MTR | 1ZSR | 2F81 | 2PK6 | 2WL0 | 3HAU | 3NU5 | 3OUB | 3SA8 | 4DQB |
| 1C6Z | 1FGC | 1N49 | 2AOC | 2F8G | 2PQZ | 2ZGA | 3HAW | 3NU6 | 3OUC | 3SA9 | 4DQC |
| 1CPI | 1FQX | 1NH0 | 2AOD | 2FGU | 2PSU | 3A2O | 3HBO | 3NU9 | 3OUD | 3SAA | 4DQE |
| 1D4H | 1G35 | 1NPA | 2AOE | 2FGV | 2PSV | 3B7V | 3HDK | 3NUJ | 3OXC | 3SAB | 4DQF |
| 1D4I | 1HBV | 1RL8 | 2AOF | 2FLE | 2PWC | 3B80 | 3HLO | 3NUO | 3PSU | 3SAC | 4DQG |
| 1D4J | 1HIV | 1SH9 | 2AOG | 2HB3 | 2PWR | 3BC4 | 3HZC | 3NWQ | 3PWM | 3ST5 | 4DQH |
| 1D4S | 1HPO | 1SP5 | 2AOH | 2HPE | 2QHY | 3BGB | 3I2L | 3NWX | 3PWR | 3T11 | 4EP2 |
| 1D4Y | 1HPV | 1T7I | 2AOI | 2HPF | 2QHZ | 3BGC | 3IA9 | 3NXE | 3QBF | 3TH9 | 4EP3 |
| 1DAZ | 1HPX | 1T7J | 2AOJ | 2I4U | 2QI0 | 3BHE | 3IAW | 3NXN | 3QIH | 3TOF | 4EPJ |
| 1DIF | 1HXW | 1TSQ | 2AVM | 2J9J | 2QI1 | 3CKT | 3K4V | 3NYG | 3QN8 | 3TOG | 4EQ0 |
| 1DMP | 1IIQ | 1TSU | 2AVQ | 2J9K | 2QI3 | 3DOX | 3KA2 | 3O99 | 3QP0 | 3TOH | 4EQJ |
| 1DW6 | 1JP5 | 1U8G | 2AZ8 | 2JE4 | 2QI4 | 3EKP | 3KDB | 3O9A | 3QPJ | 3TTP | 4EYR |
| 1EBK | 1K1T | 1UPJ | 2AZ9 | 2MIP | 2QI5 | 3EKQ | 3KDC | 3O9B | 3QRM | 3UCB | 4FE6 |
| 1EBW | 1K1U | 1W5V | 2AZB | 2NMY | 2QI6 | 3EKT | 3KDD | 3O9C | 3QRO | 3UFN | 4HVP |
| 1EBY | 1K2B | 1W5W | 2AZC | 2NMZ | 2QI7 | 3EKW | 3LZV | 3O9D | 3QRS | 3UHL | 7UPJ |
| 1EBZ | 1K2C | 1W5X | 2B60 | 2NNK | 2QNN | 3EL0 | 3MIM | 3O9E | 3R0W | 3VF5 | 9HVP |
| 1EC0 | 1KJ4 | 1W5Y | 2BPV | 2NNP | 2QNP | 3EL4 | 3MWS | 3O9F | 3R0Y | 3VF7 |  |

RT complexes

| 1C0T | 1HYS | 1JLG | 1REV | 1S6Q | 2B5J | 2OPQ | 2YNG | 3IS9 | 3KLH | 3QO9 | 4I7F |
| --- | --- | --- | --- | --- | --- | --- | --- | --- | --- | --- | --- |
| 1C0U | 1IKV | 1JLQ | 1RT3 | 1S9E | 2B6A | 2OPR | 2YNH | 3ITH | 3LAK | 3T19 | 4I7G |
| 1C1B | 1IKW | 1KLM | 1RT4 | 1S9G | 2BAN | 2OPS | 2YNI | 3JSM | 3LAL | 3T1A | 4ICL |
| 1C1C | 1IKX | 1LW0 | 1RT5 | 1SUQ | 2BE2 | 2RKI | 2ZD1 | 3JYT | 3LAM | 3TAM | 4ID5 |
| 1DTQ | 1IKY | 1LW2 | 1RT6 | 1SV5 | 2HMI | 2VG5 | 2ZE2 | 3K2P | 3LAN | 3V4I | 4IDK |
| 1DTT | 1J5O | 1LWC | 1RT7 | 1T03 | 2HND | 2VG6 | 3BGR | 3KJV | 3LP0 | 3V6D | 4IFV |
| 1EET | 1JKH | 1LWE | 1RTD | 1T05 | 2HNY | 2VG7 | 3DLE | 3KK1 | 3LP1 | 3V81 | 4IFY |
| 1EP4 | 1JLA | 1LWF | 1S1T | 1TKT | 2HNZ | 2WOM | 3DLG | 3KK2 | 3LP2 | 4G1Q | 4IG0 |
| 1FK9 | 1JLB | 1N5Y | 1S1U | 1TKX | 2I5J | 2WON | 3DOK | 3KK3 | 3MEC | 4H4M | 4IG3 |
| 1FKO | 1JLC | 1N6Q | 1S1V | 1TKZ | 2IAJ | 2YKM | 3DOL | 3KLE | 3MEE | 4H4O |  |
| 1FKP | 1JLE | 1QE1 | 1S1W | 1TL1 | 2IC3 | 2YKN | 3IG1 | 3KLF | 3QIP | 4I2P |  |
| 1HNI | 1JLF | 1R0A | 1S1X | 1TL3 | 2OPP | 2YNF | 3IRX | 3KLG | 3QLH | 4I2Q |  |
